# MoSec61β, the beta subunit of Sec61, is involved in fungal development and pathogenicity, plant immunity, and ER-phagy in *Magnaporthe oryzae*

**DOI:** 10.1080/21505594.2020.1848983

**Published:** 2020-11-29

**Authors:** Yun-Yun Wei, Shuang Liang, Yun-Ran Zhang, Jian-Ping Lu, Fu-Cheng Lin, Xiao-Hong Liu

**Affiliations:** a State Key Laboratory for Managing Biotic and Chemical Treats to the Quality and Safety of Agro-products, Institute of Biotechnology, Zhejiang University, Hangzhou, China; b Zhejiang Provincial Laboratory of Life Sciences and Biomedicine, Key Laboratory of Structural Biology of Zhejiang Province, School of Life Sciences, Westlake University, Hangzhou, China; c Institute of Basic Medical Sciences, Westlake Institute for Advanced Study, Hangzhou, China; d College of Life Sciences, Zhejiang University, Hangzhou, China; e State Key Laboratory for Managing Biotic and Chemical Treats to the Quality and Safety of Agro-products, Institute of Plant Protection and Microbiology, Zhejiang Academy of Agricultural Sciences, Hangzhou, China

**Keywords:** Sec61β, development, pathogenesis, plant immunity, ER-phagy

## Abstract

The process of protein translocation into the endoplasmic reticulum (ER) is the initial and decisive step in the biosynthesis of all secretory proteins and many soluble organelle proteins. In this process, the Sec61 complex is the protein-conducting channel for transport. In this study, we identified and characterized the β subunit of the Sec61 complex in *Magnaporthe oryzae* (MoSec61β). Compared with the wild-type strain Guy11, the Δ*Mosec61β* mutant exhibited highly branched mycelial morphology, reduced conidiation, high sensitivity to cell wall integrity stress, severely reduced virulence to rice and barley, and restricted biotrophic invasion. The turgor pressure of Δ*Mosec61β* was notably reduced, which affected the function of appressoria. Moreover, Δ*Mosec61β* was also sensitive to oxidative stress and exhibited a reduced ability to overcome plant immunity. Further examination demonstrated that MoSec61β affected the normal secretion of the apoplastic effectors Bas4 and Slp1. In addition, Δ*Mosec61β* upregulated the level of ER-phagy. In conclusion, our results demonstrate the importance of the roles played by MoSec61β in the fungal development and pathogenesis of *M. oryzae*.

## Introduction

*Magnaporthe oryzae* is the most important pathogen infecting rice, wheat, and other grass species[1]. *M. oryzae* infection begins after the conidia are spread by wind or dew drops to the host surface. Under proper conditions, the conidium germinates and forms a polarized germ tube. Next, the tip of the germ tube differentiates into a dome-shaped infection structure called the appressorium [2]. Appressorium development is accompanied by energy recycling in which the nucleus, glycogen and lipid droplets in the conidia are degraded by autophagy or transported to the appressorium [3]. After the appressorium matures, it forms a penetration peg to rupture the cuticle of the host through glycerol-derived hydrostatic pressure. Subsequently, the filamentous primary invasive hyphae (IH) extend inside the cell and develop into the bulbous secondary invasive hyphae for transcellular infection. Colonization by invasive hyphae results in the appearance of necrotic lesions.

**Figure 1.**
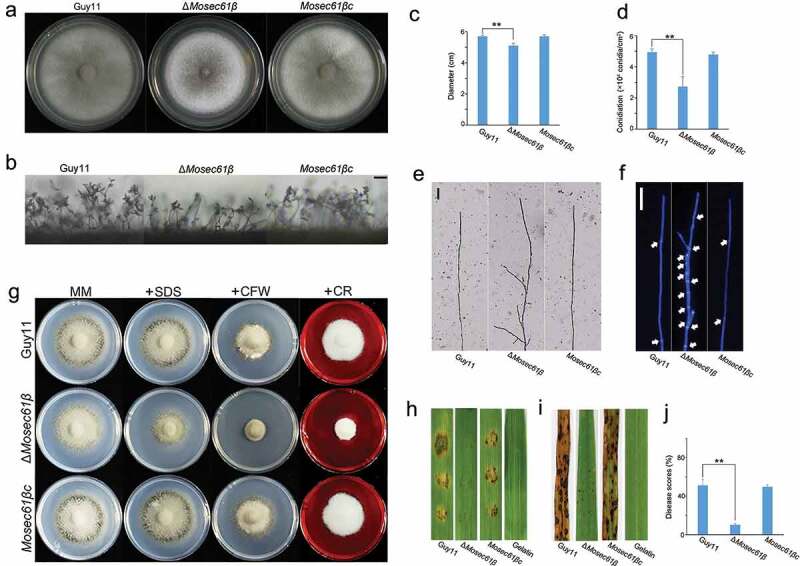
MoSec61β is involved in hyphal growth, asexual reproduction, cell wall integrity, and pathogenicity. (a) Growth of Guy11, Δ*Mosec61β*, and *Mosec61β*c on CM plates. Strains were inoculated on CM plates for 8 days. (b) Conidiophores of wild-type, Δ*Mosec61β*, and *Mosec61β*c. Bar = 50 μm. (c) Mycelial diameters of the wild-type, Δ*Mosec61β*, and *Mosec61β*c strains. (d) Conidiation of Guy11, Δ*Mosec61β*, and *Mosec61β*c. (e) Morphology of vegetative hyphae of Guy11, Δ*Mosec61β*, and *Mosec61β*c on cover glass. Bar = 50 μm. (f) Vegetative hyphae of wild-type, Δ*Mosec61β*, and *Mosec61β*c stained with CFW. White arrows point to the septa. Bar = 20 μm. (g) Strains were incubated on MM plates supplemented with various stress inducers at 25°C for 7 days. Growth of strains in media supplemented with 0.0025% SDS, 100 μg/mL CFW, and 50 μg/mL Congo red (CR). (h) Pathogenicity on barley leaves. Twenty microliters conidial drops (5 × 10^4^ mL^−1^) were inoculated on barley leaves. Photographs were taken after 4 dpi. (i) Pathogenicity of rice seedlings. Conidia (5 × 10^4^ mL^−1^) were sprayed on 21-day-old rice seedlings. Photographs were taken after 7 dpi. (j) Disease score assays for Guy11, Δ*Mosec61β*, and *Mosec61β*c. The proportion of lesion areas in 5-cm leaves was measured by Photoshop CS6. Error bars represent the standard deviation. Signiﬁcant differences between the mutant and wild-type strains, as estimated by Duncan’s test: **P < 0.01, *P < 0.05

**Figure 2.**
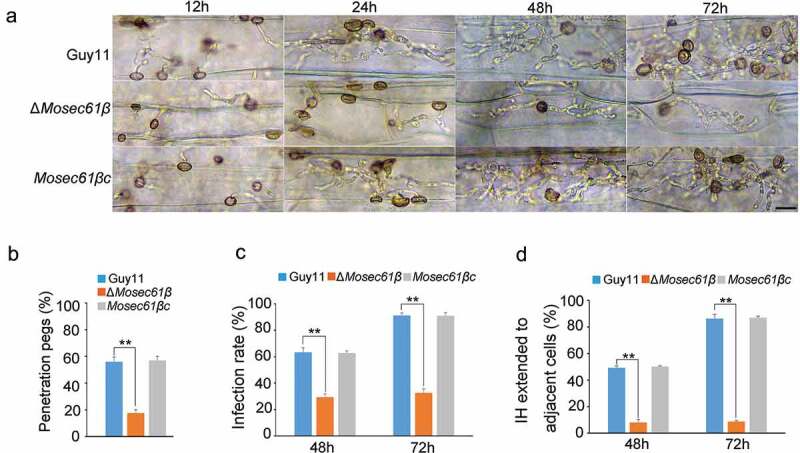
MoSec61β is required for plant penetration and invasive growth. (a) Penetration assays on barley leaves were performed after 12, 24, 48, and 72 hpi. Bar = 50 μm. (b) The rate of penetration peg formation of Guy11, Δ*Mosec61β*, and *Mosec61β*c. (c) Penetration rate of appressoria at 48 hpi and 72 hpi. (d) The percentage of invasive hyphae extended to adjacent cells at 48 hpi and 72 hpi. Error bars represent the standard deviation. Signiﬁcant differences between the mutant and wild-type strains, as estimated by Duncan’s test: **P < 0.01, *P < 0.05

**Figure 3.**
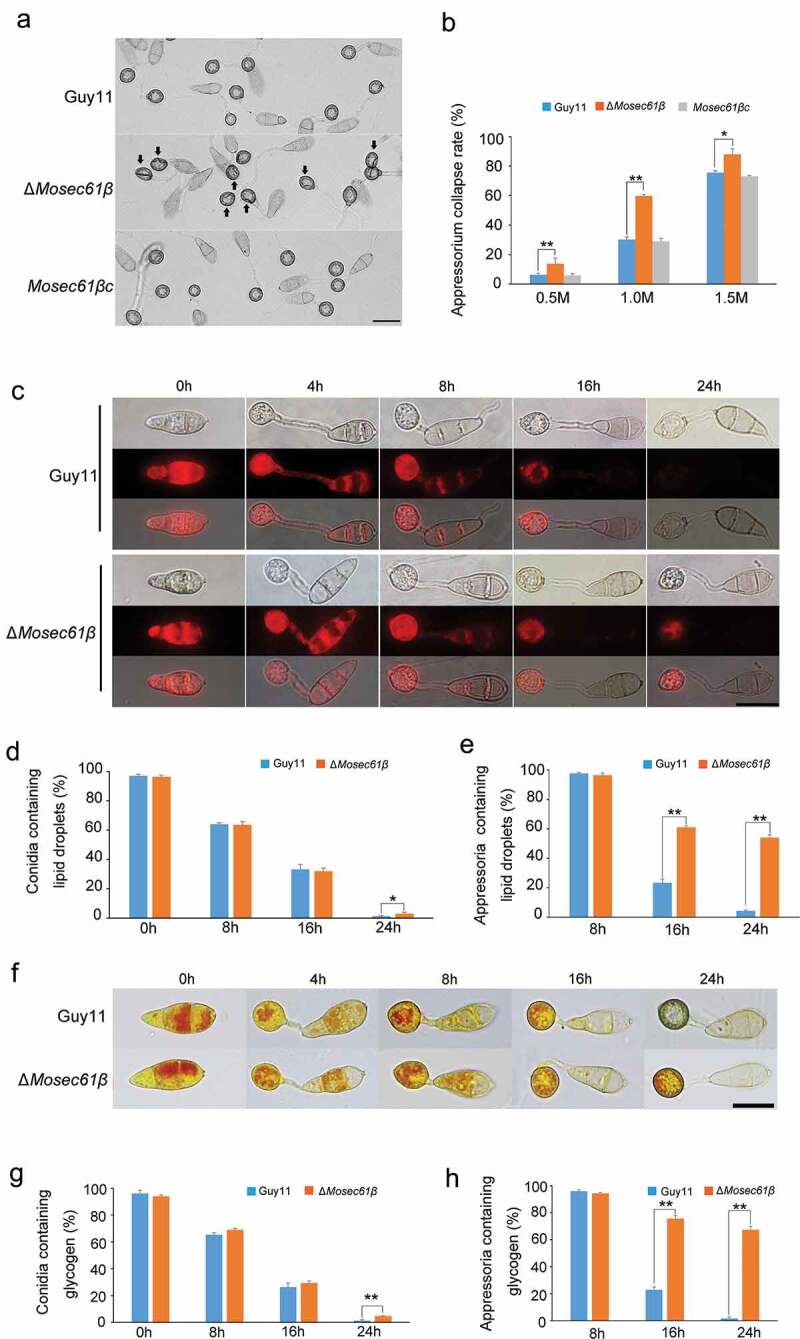
(a) Collapse of appressoria at 0.5 M glycerol. 5 Bar = 20 μm. (b) A 0.5–1.5 molar concentration of glycerol solution was applied to examine the collapse rate of appressoria in Guy11, Δ*Mosec61β*, and *Mosec61β*c. Arrows indicate the collapsed appressoria. (c) Cellular distribution of lipid droplets during appressorium development. Samples were stained with Nile red and observed with UV epiﬂuorescence. The lipid droplets show a red signal ﬂuorescence. Bar = 20 μm. (d) The percentage of conidia containing lipid droplets during appressorial development. (e) The percentage of appressoria containing lipid droplets. (f) Cellular distribution of glycogen during appressorium development. Bar = 20 μm. Samples were stained with KI/I_2_ solution at the indicated time phase. The glycogen appears as dark brown deposits. (g) The percentage of conidia containing glycogen. (h) The percentage of appressoria containing glycogen during appressorium development. Error bars represent the standard deviation. Significant differences compared with the wild-type strain were estimated by Duncan’s test: **P < 0.01, *P < 0.05

**Figure 4.**
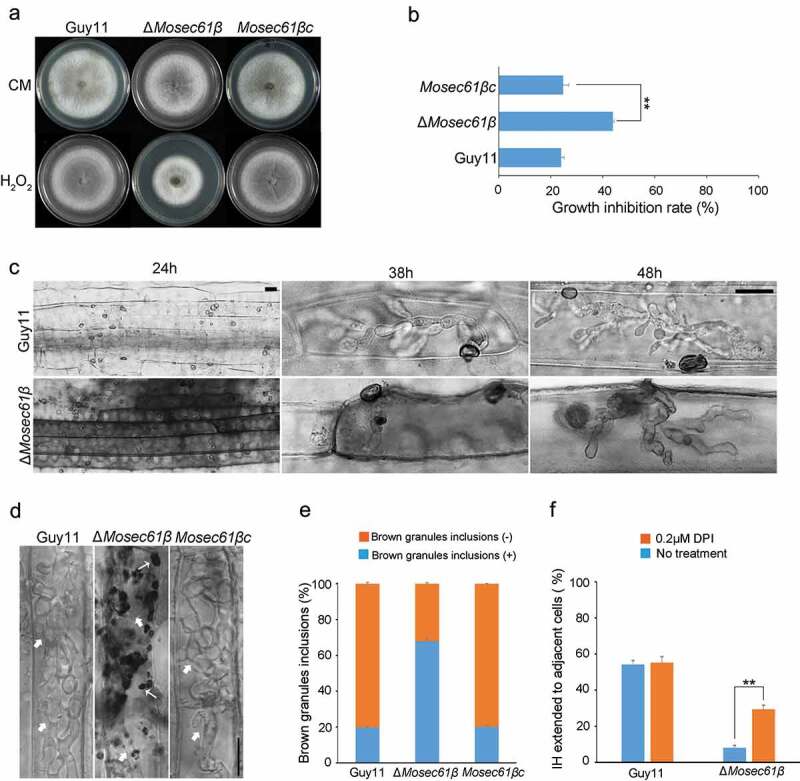
Δ*Mosec61β* is sensitive to oxidative stress and cannot scavenge ROS. (a) *M. oryzae* strains grown on 5.0 mM H_2_O_2_. (b) Relative growth of mycelial colonies on 5.0 mM H_2_O_2_. (c) Reactive oxygen species (ROS) capture by 3,30-diaminobenzidine (DAB) staining in infected barley leaves. Bar = 20 μm. (d) ROS capture by DAB staining in infected rice leaf sheaths at 48 hpi. White thick arrows point to the invasive hyphae, white thin arrows point to orange brown granules. Bar = 20 μm. (e) The percentage of *M. oryzae* strains that can induce brown granules. (f) Percentage of appressorium-mediated penetration and infectious hyphae development of Guy11 and Δ*Mosec61β* in DPI-treated rice sheaths. Error bars represent the standard deviation. Significant differences compared with the wild-type strain were estimated by Duncan’s test: **P < 0.01, *P < 0.05

**Figure 5.**
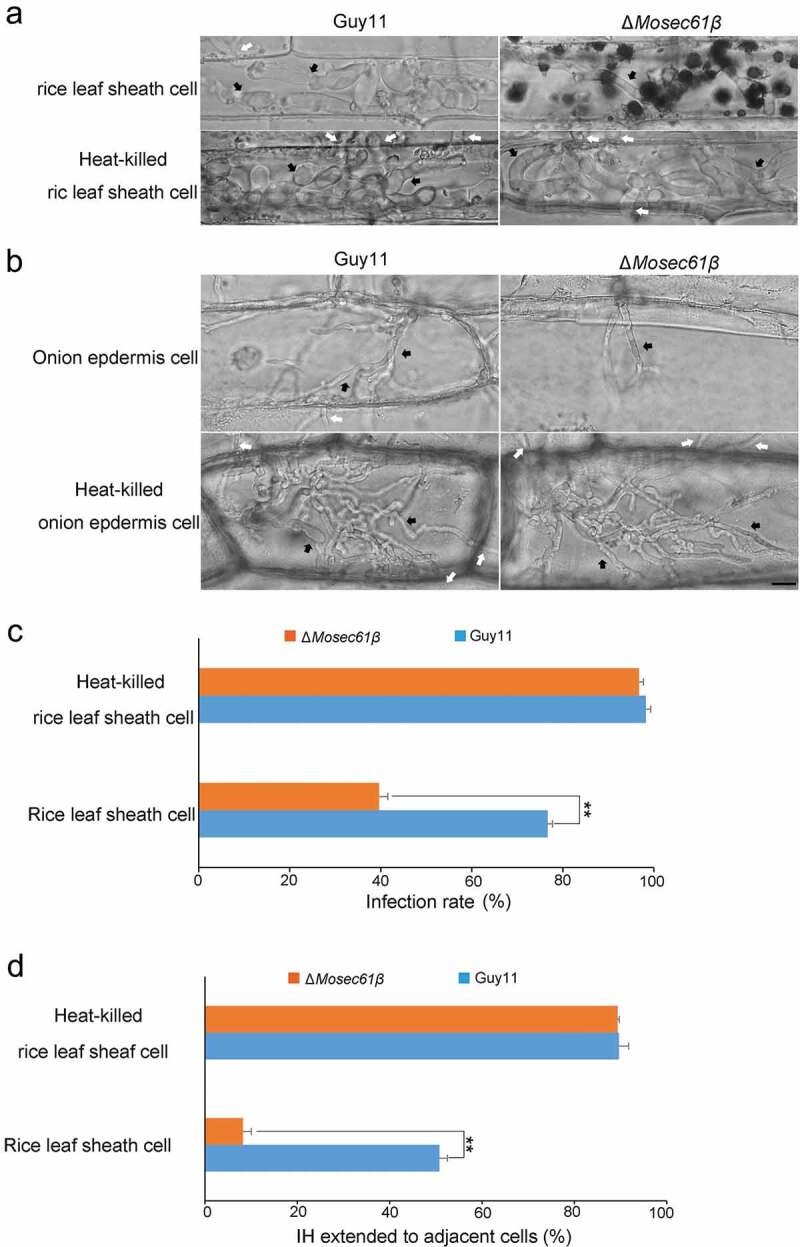
Penetration assays with heat-killed rice leaf sheaths and onion epidermis. (a) Conidia solution was inoculated into normal or heat-treated leaf sheaths, and infection was observed after 48 hpi. Bar = 20 μm. Black arrows point to IH. White arrows point to IH, which was expanded in adjacent cells. (b) Conidia solution was inoculated into normal or heat-treated onion epidermal cells, and infection was observed after 48 hpi. Bar = 20 μm. Black arrows point to IH. White arrows point to IH, which was expanded in adjacent cells. (c) Statistical analysis of the infection rate of the appressoria of the *M. oryzae* strains in leaf sheaths at 48 hpi. (d) The percentage of invasive hyphae extended to adjacent cells in normal or heat-treated leaf sheaths at 48 hpi. Error bars represent the standard deviation. Significant differences compared with the wild-type strain were estimated by Duncan’s test: **P < 0.01, *P < 0.05

**Figure 6.**
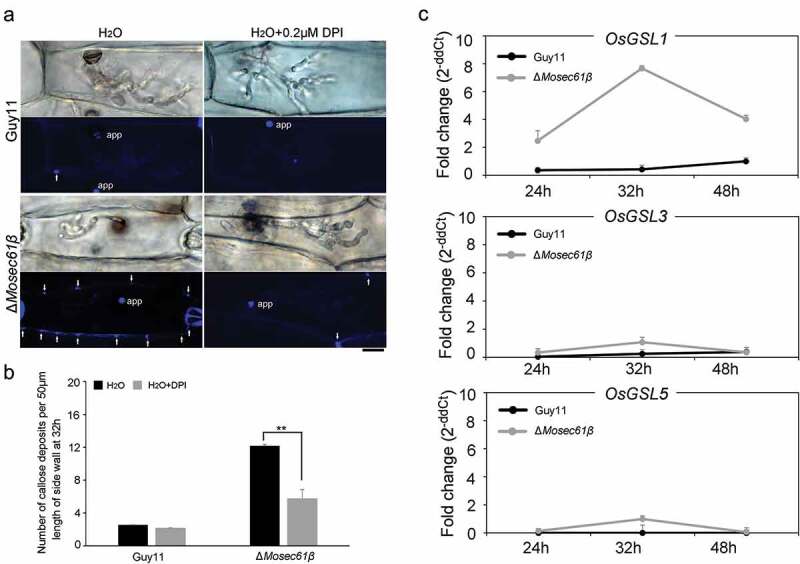
Callose deposition in barley leaves. (a) Deposited callose in leave cells under different treatment. Arrows indicate the callose depositions. app: appressorium. Bar = 10 μm. (b) The number of callose deposition per 50 μm length of side wall at 32 h. Error bars represent the standard deviation. Significant differences compared with the wild-type strain were estimated by Duncan’s test: **P < 0.01, *P < 0.05. (c) The expression level of callose synthase-encoding genes (*OsGSL1, OsGSL3*, and *OsGSL5*) in Guy11 and Δ*Mosec61β-*challenged rice leaves

**Figure 7.**
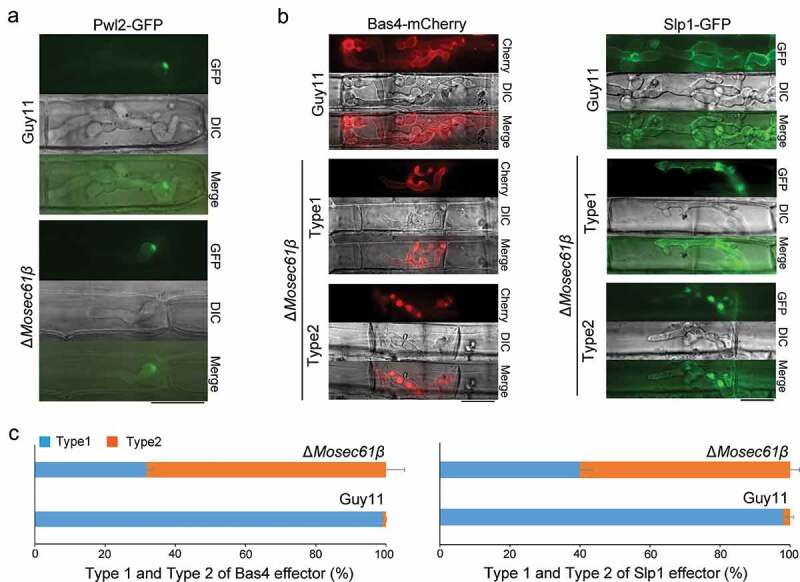
Distribution of effectors in IH of *M. oryzae* strains. (a) Fluorescence localization of the Pwl2 cytoplasmic effector in a rice sheath infected with *M. oryzae*. Bar = 20 μm. (b) Distribution of the apoplastic effectors Bas4 and Slp1 in wild-type and Δ*Mosec61β*. Bar = 20 μm. (c) Statistical analysis of the localization of the two types of fluorescent effectors in *M. oryzae*. Error bars represent the standard deviation

**Figure 8.**
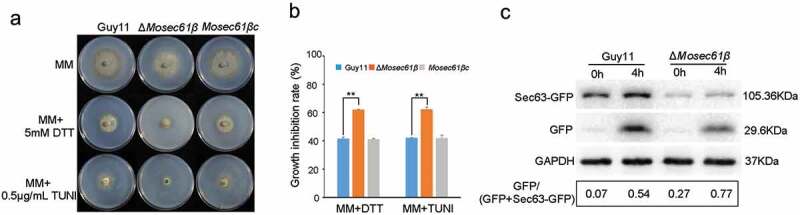
Responses of *M. oryzae* strains to ER stress. (a) Mycelial colonies of Guy11, Δ*Mosec61β*, and *Mosec61β*c cultured on MM media containing 0.5 μg/mL TUNI and 5.0 mM DTT at 25°C with a 16 h light and 8 h dark cycle for 8 days. (b) Growth inhibition rate of mycelial colonies on 0.5 μg/mL TUNI and 5.0 mM DTT. (c) ER-phagy of Sec63-GFP in Guy11 and Δ*Mosec61β*. Total proteins were extracted from the Sec63-GFP expressed strains exposed to nitrogen starvation conditions with 5 μM DDT for 0 and 4 h. Full-length Sec63-GFP and free GFP were detected using GFP antibodies as described in the Materials and Methods. The extent of ER-phagy was estimated by calculating the amount of free GFP compared with the total amount of intact Sec63-GFP and free GFP. Quantitative analysis of the individual bands was performed using ImageJ software. Error bars represent the standard deviation. Signiﬁcant differences between the mutant and wild-type strains, as estimated by Duncan’s test: **P < 0.01, *P < 0.05

To respond to pathogen stimulation and invasion, plants have evolved an advanced and complex immune system that is sensitive to stimuli from the outside environment [4]. Recognition of pathogen-associated molecular patterns (PAMPs) by pattern recognition receptors (PRRs) leads to PAMP-triggered immunity (PTI) [5]. Furthermore, when PTI is no longer protective of plants, resistance (R) proteins recognize pathogen-derived effectors and initiate effector-triggered immunity (ETI) [6]. The two immune processes share common defense responses, including an outburst of reactive oxygen species (ROS), secretion of antimicrobial compounds, and reinforcement of plant cell walls [7]. In addition to the plant’s immune response, there is also evidence that pathogens interfere with plant immunity. Two bio-structures are formed where pathogens secrete effectors in response to plant immunity. Host-derived extrainvasive hyphal membranes (EIHM) are formed to enfold biotrophic IH [8], and host membrane-rich structures called biotrophic interfacial complexes (BIC) are formed focally at the periphery of invasive hyphae during biotrophic invasion [9]. Additionally, apoplastic and cytoplasmic effectors, two distinct effector secretion systems, have been identified in *M. oryzae* [10]. Apoplastic effectors are associated with host plants before cytoplasmic effectors and partly decide the outcome of the interaction between pathogens and plants [11]. The apoplastic effector Bas4 and Slp1 are dispersed in the extracellular compartment formed by EIHM and the IH membrane to indirectly act on host plant cells through signal transduction [7,12]. The apoplastic effectors are secreted via the conserved ER (endoplasmic reticulum)-to-Golgi secretion pathway. Conventional protein secretion is the transport route of secretory proteins from the endoplasmic reticulum (ER) to the Golgi apparatus (GA) and subsequently through secretory vesicles or secretory particles into the plasma membrane (PM) [13]. Conversely, the function of cytoplasmic effectors is to impair the normal physiological metabolism inside plant cells [14]. Pwl2 is a classical cytoplasmic effector that preferentially accumulates in BIC after being secreted from IH and subsequently enters the plant host cell [15]. The delivery of cytoplasmic effectors is mediated by the exocyst complex, which is essential for establishing epithelial polarity, morphogenesis, and homeostasis [10,16].

The Sec61 complex is a membrane channel on the ER that is involved in the translocation of newly synthesized precursor polypeptides into the ER lumen or on the ER membrane [17]. Sec61 is a highly conserved multisubunit protein complex that consists of three subunits, Sec61α, Sec61β, and Sec61γ, in eukaryotic cells. In budding yeast, the core proteins are Sec61, Sbh1, and Sss1. The α subunit forms the pore channel through which a polypeptide chain passes [18], whereas the γ subunit stabilizes the protein translocation [19]. Unlike Sec61α and Sec61γ, whose functions have been thoroughly investigated, the function of the β-subunit is still under study. At present, it is known that Sec61β (Sbh1 and Sbh2) is not necessary for the functional integrity of Sec61 in yeast, although it does promote the process of protein translocation [20]. In contrast to the results obtained in *Saccharomyces cerevisiae*, Sec61β has been determined to be essential for the embryogenesis of *Drosophila melanogaster* [21]. In addition, a study on barley resistance suggested that Sec61β is required for plant susceptibility to powdery mildew [22]. However, although studies have highlighted the importance of Sec61β, the mechanism by which this protein functions has not been elucidated. To the best of our knowledge, the functions of the Sec61 complex in plant pathogenic fungi have not been studied to date.

In this study, we identified the beta subunit of Sec61 in *M. oryzae*, designated MoSec61β. Deletion of *MoSEC61β* was associated with abnormal polarized hyphal growth, reduced conidiation and appressorium turgor pressure, defects in cell wall integrity, attenuated utilization of glycogen and lipid droplets, and weak virulence to rice and barley. Furthermore, penetration assays revealed that the ability to form penetration pegs was impaired in Δ*Mosec61β*. Although a small number of penetration pegs are formed successfully, the IH still fails to colonize adjacent cells because it no longer has the capacity to eliminate the host ROS. Meanwhile, high expression level of pathogenicity-related gene *PR1a* in leaves of Δ*Mosec61β-*challenged rice, and weak capacity in degrading callose of Δ*Mosec61β* suggested the essential role of MoSec61β in overcoming plant defense responses. Additionally, the distribution of the apoplastic effector Bas4 and Slp1 were disrupted in Δ*Mosec61β*, indicating the important role played by *MoSEC61β* in ER-to-Golgi transport. Also, the deletion of MoSec61β leads to more intense ER-phagy. In conclusion, we demonstrate the biological function of the β subunit of the Sec61 complex in *M. oryzae* and provide new evidence for ER-mediated ER-phagy and apoplastic effector secretion in plant pathogenic fungi.

## Results

### *MoSec61β is essential for conidiogenesis, polarized hyphal growth, cell wall integrity, and virulence of* M. oryzae

Sequence alignment analysis showed that MGG_03644 shared 48.72% and 57.83% identity with *S. cerevisiae* Sbh1p and Sbh2p, respectively (Fig. S1). We termed MGG_03644 as *MoSEC61β*. MoSec61β is predicted to encode 144 amino acids (aa) and spans the membrane once (85–103 aa), with the N-terminus being in the cytosol. Then, the biological functions of MoSec61β were explored using target gene replacement. In addition, the full-length genomic copy of *MoSEC61β* was reintroduced into Δ*Mosec61β* for genetic complementation analysis, and we named the complementation strain *Mosec61βc*.

The growth rate of Δ*Mosec61β* is lower than that of the wild-type Guy11 and the complementation strain *Mosec61βc* (Figure 1(a,c)). Microscopic observation showed that the pear-shaped conidia of Guy11, Δ*Mosec61β*, or *Mosec61βc* were distributed in a concentric axis at the top of the fascicular conidiophore (Figure 1(b)). However, statistical analysis showed that the number of conidia produced by Δ*Mosec61β* was clearly decreased. Compared to the wild-type strain, the sporulation in Δ*Mosec61β* was decreased to half of that in Guy11 (Figure 1(d)). Additionally, compared with the wild-type Guy11 and the complementation strain *Mosec61βc*, the Δ*Mosec61β* mycelia produced more apical and subapical branches, and the wild-type Guy11 and *Mosec61βc* maintained the vertical growth of mycelium without sub-top branches (Figure 1(e)). To clearly observe the morphology of hyphae, we stained the mycelia of Guy11, Δ*Mosec61β* and *Mosec61βc* using calcofluor white (CFW). The mycelia of wild-type Guy11 and *Mosec61βc* were straight, and the cell intervals were largely equidistant (Figure 1(f)). In contrast, the interval of Δ*Mosec61β* mycelia had irregular branches, and the spacing interval of each cell was shorter than that of Guy11 and was not evenly spaced (Figure 1(f)). MoSec61β was determined to be involved in the morphogenesis and separation of vegetative mycelia.

To identify the role of MoSec61β in cell wall integrity, we monitored the effects of various cell wall perturbing agents on the Δ*Mosec61β* mutant. Mycelial growth was measured on MM plates supplemented with CFW, Congo red (CR) and SDS (sodium dodecyl sulfate), compounds known to cause cell wall stress. As shown in Figure 1(g), the sensitivity of the Δ*Mosec61β* mutant to CFW and Congo red was significantly different from that of both the wild-type Guy11 and the complementation strain *Mosec61βc*. The growth inhibition rates of Δ*Mosec61β* to CFW and Congo red were significantly different from those of Guy11 and *Mosec61βc* (Fig. S2). These results indicated that the loss of MoSec61β affects cell wall integrity.

To investigate whether MoSec61β is related to the virulence of *M. oryzae*, two susceptible hosts, rice (CO-39) and barley (ZJ-8), were employed for the assessment of the pathogenicity of strains. After inoculation on barley leaves with conidia suspension (5 × 10^4^ conidia/ml) for 4 days, the wild-type Guy11 and the complementation strain *Mosec61βc* caused yellow and brown lesions with rotten plant tissues, while inoculation with Δ*Mosec61β* resulted in tiny lesions (Figure 1(h)). Similarly, when inoculated onto barley leaves with mycelium plugs, Δ*Mosec61β* also caused weaker disease lesions compared to the wild-type Guy11 and the complementation strain *Mosec61βc* (Fig. S3). When sprayed onto 21-day rice seedlings with conidia suspension (5 × 10^4^ conidia/ml), Δ*Mosec61β* caused small necrotic flecks, while the wild-type Guy11 and the complementation strain *Mosec61βc* caused typical spindle-like, gray centered blast lesions and many merged lesions (Figure 1(i)). The disease lesion areas in 5-cm-long infected leaves caused by Δ*Mosec61β* (10.23 ± 1.23%) were significantly smaller than those caused by the wild-type Guy11 (51.30 ± 4.87%) and the complementation strain *Mosec61βc* (49.75 ± 1.96%) 7 days post-inoculation (dpi) (Figure 1(j)). Thus, MoSec61β was observed to play an important role in pathogenicity.

### *MoSec61β is involved in the development of invasive hyphae in* M. oryzae

To determine the reasons for the reduced virulence of Δ*Mosec61β*, we first analyzed conidium germination and appressorium formation. However, there were no significant differences in conidia germination, appressorium formation, or appressorium morphology among Guy11, Δ*Mosec61β*, and *Mosec61βc* (Table S1, Fig. S4). Then, we performed penetration assays on barley leaves. Few appressoria of Δ*Mosec61β* could form penetration pegs (Figure 2(a,b)). Additionally, the appressorial penetration rate of Δ*Mosec61β* on barley leaves was significantly decreased compared to that of the wild-type and complementation strains (Figure 2(a)). At 48 hpi (hours post-inoculation), the appressorial penetration rate of Δ*Mosec61β* was approximately half of that in the wild-type and complementation strains *Mosec61βc* (Figure 2(c)). At 72 hpi, more than 90% of Guy11 appressoria penetrated barley cells. However, only 30% of the appressoria of Δ*Mosec61β* penetrated barley cells (Figure 2(c)). Further observation showed that Δ*Mosec61β* displayed defects in IH extension. At 48 hpi, nearly 50% of IH in the wild-type and *Mosec61βc* strains showed transcellular infection, and less than 10% IH of Δ*Mosec61β* invaded neighboring cells (Figure 2(d)). At 72 hpi, more than 80% of IH in the wild-type and *Mosec61βc* strains colonized other cells, but only 10% of IH in Δ*Mosec61β* expanded into adjacent cells (Figure 2(d)). These data demonstrated that the infection and IH extension of Δ*Mosec61β* are notably impaired.

### MoSec61β affects turgor pressure in appressoria and mobilization of glycogen and lipid droplets from conidia to appressoria

The process of penetration mediated by the appressorium requires a large amount of inner turgor pressure, which can enable the appressorium to produce adequate mechanical strength and facilitate rupture of the host cuticle with narrow-penetration hyphae [23]. As described previously, most of the appressoria of Δ*Mosec61β* cannot form penetration pegs, and we investigated if the appressorium turgor pressure of Δ*Mosec61β* was impaired. Specifically, we performed an incipient cytorrhysis assay to measure the turgor pressure exerted by mature appressoria. In this assay, a 0.5–1.5 M concentration of glycerol solution was applied to examine the collapse rate of appressoria. Under treatment with 1 M glycerol, approximately 30% of 24 h appressoria of the wild-type strain and the complementation strain *Mosec61βc* collapsed. However, 60% of Δ*Mosec61β* collapsed with this treatment (Figure 3(a,b)). With the increase of glycerol concentration to 1.5 M, the collapse rate of Δ*Mosec61β* appressoria increased to nearly 90% and was higher than that of the wild-type and *Mosec61βc* strains (Figure 3(b)). It was concluded that the turgor pressure of the Δ*Mosec61β* appressoria was decreased compared with that of the wild-type strain.

The glycerol in appressoria is primarily produced by the transfer and utilization of glycogen and lipids in conidia. To investigate the glycogen distribution and lipid turnover of Δ*Mosec61β*, we employed potassium iodide to stain glycogen and employed Nile red to stain lipids during appressorium development. From 0 to 4 h in the preliminary stage of appressorium development on a hydrophobic surface, abundant lipids were seen in conidia and appressoria of Guy11 and Δ*Mosec61β* (Figure 3(c)). At 8 hpi, more than 60% lipids in conidia of the wild-type Guy11 and Δ*Mosec61β* strains were transported to appressoria. At 24 hpi, a significantly higher proportion of Δ*Mosec61β* conidia contained lipids (Figure 3(d)). Compared to Guy11, more than 60% of Δ*Mosec61β* appressoria contained lipids at 16 and 24 hpi (Figure 3(e)). Similarly, the distribution of glycogen exhibited the same pattern as lipids in Δ*Mosec61β*, as shown in Figure 3(f–h). The above results indicate that *MoSEC61β* is required for lipid droplets and glycogen mobilization from conidia to appressoria.

### *Ability to scavenge host ROS is reduced in Δ*Mosec61β

When plants are attacked by pathogens, the plant cells generate massive reactive oxygen species (ROS) to inhibit or directly kill invading pathogens [24]. We hypothesize that the reason Δ*Mosec61β* IH failed to colonize adjacent cells was related to its abnormal ability to scavenge ROS. To confirm this possibility, we determined the ability of Δ*Mosec61β* to resist redox stress. The mycelial plugs of Guy11, Δ*Mosec61β*, and *Mosec61βc* were inoculated onto CM supplemented with the oxidative stress agent H_2_O_2_ (5 mM) (Figure 4(a)). The growth inhibition rate of Δ*Mosec61β* was significantly higher than that of Guy11 (Figure 4(b)). This result indicates that the Δ*Mosec61β* mutant is more sensitive to oxidative stress than the wild-type strain.

Then, 3,3ʹ-diaminobenzidine (DAB) staining was used to capture hydrogen peroxide (one type of ROS) produced in the infected barley leaves. At 38 hpi, Guy11 was able to invade the surface cells and was observed to expand to the surrounding cells, and no obvious ROS were dyed. In contrast, Δ*Mosec61β* IH was confined in the first cell and surrounded by obvious reddish-brown dyed ROS (Figure 4(c)). Similar results were obtained when the strains were incubated on a rice sheath (Figure 4(d)). There was an abundant accumulation of dark brown granules among IH of Δ*Mosec61β* (67.88 ± 1.50%) in rice cells. However, few brown granules (19.78 ± 0.19%) were observed in the wild-type Guy11 and the complementation strain (Figure 4(e)). The dark brown granules indicated the immune response of rice, further demonstrating that the capacity of Δ*Mosec61β* to scavenge host ROS was reduced.

Furthermore, an inhibitor of NADPH oxidase diphenyleneiodonium (DPI) was applied to the rice sheath to determine whether ROS accumulation is the primary factor causing IH defects. Under normal conditions (without DPI), less than 10% of the appressoria of Δ*Mosec61β* penetrated into adjacent cells at 48 h, but more than 50% of the wild-type appressoria successfully developed IH in the neighboring cells. Under the condition of 0.2 μM DPI treatment, the extension of Δ*Mosec61β* IH to adjacent cells increased threefold at 48 h. However, there were no obvious changes in the wild-type strain (Figure 4(f)). These data confirmed that the attenuated biotrophic growth of Δ*Mosec61β* was due to host ROS accumulation.

### *Δ*Mosec61β *is defective in overcoming plant defense responses*

To further confirm that the defects of Δ*Mosec61β* in infectious growth were caused by the failure to resist the immunity of plants, we used pathogens to infect heat-killed plant cells (Figure 5(a)). The penetration assay was carried out with rice leaf sheaths incubated at 75°C for 25 min before inoculation. After 48 hpi, almost all of the appressoria formed by the wild type (98.24 ± 1.04%) and Δ*Mosec61β* (96.73 ± 0.97%) penetrated the epidermal cells of rice leaf sheaths (Figure 5(c)). In addition, it is obvious that the Δ*Mosec61β* IH are no longer confined in the first cell, and the percentage of IH extending to the neighboring cells is greatly increased. There was no significant difference compared with the wild-type Guy11 (90.14 ± 2.30%) and the Δ*Mosec61β* mutant (89.63 ± 2.10%) (Figure 5(d)). We reperformed the penetration assays with heat-killed onion epidermal cells, and similar infectious patterns were observed in the wild-type and Δ*Mosec61β* strains (Figure 5(b)).

Pathogenesis-related (PR) proteins have been used as markers of plant defense responses. In order to further investigate whether the plant defense genes were stimulated by infection with Δ*Mosec61β*, the expression patterns of *PBZ1* and *PR1a* genes were analyzed through quantitative RT-PCR. *PBZ1*, a probenazole-inducible gene from rice, triggers non-race specific resistance in rice plants against rice blast fungus [25]. *PR1a* is one of the PR proteins, which accumulates after blast fungus infection in rice [26]. The expression level of *PBZ1* was up-regulated in leaves of blast-fungus-challenged rice at 24 hpi, 32 hpi, and 48 hpi, whereas there were no obvious differences in Δ*Mosec61β* or Guy11-challenged rice leaves (Fig. S5a). In contrast, the expression level of *PR1a* in leaves of Δ*Mosec61β-*challenged rice was higher than that in leaves of Guy11-challenged rice at 24 hpi and 48 hpi (Fig. S5B). The expression level of *PR1a* in leaves of Δ*Mosec61β-*challenged rice at 24 hpi was 4 folds as high as that in leaves of Guy11-challenged rice (Fig. S5b). The induction of plant defense responses in Δ*Mosec61β*-challenged rice may contribute to the retardation of IH development.

Callose, a β-1,3-glucanase induced by plant defense responses, provides chemical defenses at the cellular sites of attack [27]. To investigate the induction of callose by infection of the Δ*Mosec61β* mutant and the wild-type Guy11 respectively, we observed the accumulation of callose deposits at the cell wall crossing sites around IH using aniline blue staining. As shown in Figure 6(a), the number of the callose deposits around the Δ*Mosec61β* IH was significantly more than that around the Guy11 IH at 32 dpi. Treatment of DPI decreased the number of the callose deposits around the Δ*Mosec61β* IH (Figure 6(a,b)). Meanwhile, the expression levels of 3 callose synthase-encoding genes (*OsGSL1, OsGSL3, OsGSL5*) were investigated using quantitative RT-PCR. The expression level of *OsGSL1* reached the peak at 32 hpi in the leaves of Δ*Mosec61β-*challenged rice, and it was 20 folds as high as that in the leaves of wild-type Guy11-challenged rice. In addition, the expression of *OsGSL3* and *OsGSL5* genes was also slightly induced in leaves of the Δ*Mosec61β-*challenged rice, with higher induction levels as compared to those in leaves of the Guy11-challenged rice at 32 hpi (Figure 6(c)). These data indicated that Δ*Mosec61β* was defective in overcoming plant defense responses because of its weak capacity in degrading callose. The above results suggested that MoSec61β is necessary for overcoming plant defense responses.

### *Δ*Mosec61β *partially disrupts the localization of the apoplastic effectors Bas4 and Slp1*

To assess whether the MoSec61β protein is involved in the secretion of effectors, we performed live cell imaging of biotrophic invasion by transformants expressing the cytoplasmic effector fusion protein Pwl2-GFP and the apoplastic effector fusion protein Bas4-mCherry, Slp-GFP. During the early infection stage at 48 hpi, Pwl2-GFP localized to a single punctate BIC in the wild-type Guy11 and the Δ*Mosec61β* mutant (Figure 7(a)), indicating that secretion of cytoplasmic effectors was not impaired in Δ*Mosec61β*. In wild-type Guy11, the Bas4-mCherry fusion protein outlines the IH and occupies an inner layer of the BIC. In contrast, the localization of Bas4-mCherry for the Δ*Mosec61β* mutant was partially disrupted, while localization of the Pwl2-GFP fusion appeared to be normal. Localization patterns of Bas4-mCherry in the Δ*Mosec61β* mutant could be further divided into two types, as shown in Figure 7(b). With type 1, the fluorescence of Bas4-mCherry (32%) was uniformly wrapped the IH and was located in EIHM, which is the same as with the wild-type strain. In type 2, the localization of Bas4-mCherry could not be correctly accumulated in EIHM (68%). In contrast, nearly all randomly imaged infection sites of the wild-type strain showed the type 1 pattern (Figure 7(c), Bas4-mCherry). Similar results were observed in apoplastic effector Slp1 (Figure 7(c), Slp1-GFP). About 98% of Slp1-GFP could trace out IH and locate in EIHM in the wild-type Guy11. In contrast, two types of Slp1-GFP localization patterns were also observed in the Δ*Mosec61β* mutant, type 1 (~ 40% of Slp1-GFP could be correctly accumulated in EIHM) and type2 (~ 60% could not be correctly accumulated in EIHM) (Figure 7, Slp1-GFP). These results confirmed that the localization patterns of Bas4-mCherry and Slp1-GFP were seriously impaired in Δ*Mosec61β*. As previously described, MoSec61β is an ER transmembrane protein, and its deletion may cause abnormal secretion of effector proteins. These results indicate that proper localization of apoplastic effectors in EIHM depends in part on MoSec61β.

### MoSec61β negatively regulates ER-phagy

In our study, Δ*Mosec61β* exhibited defects similar to those of the atg-deficient mutants, such as reduced turgor pressure, retarded utilization of lipids and glycogen, and reduced virulence. However, neither conidia nuclei degradation nor GFP-MoAtg8 degradation under nutritional deprivation conditions showed the dysregulated autophagy process (Fig. S6, S7). Autophagic cell death and macroautophagy were not impaired in Δ*Mosec61β*. Interestingly, we unexpectedly observed that the growth of Δ*Mosec61β* is sensitive to ER stress under the ER stress factors tunicamycin (TUNI) or dithiothreitol (DTT) (Figure 8(a)). Under treatment with 0.5 μg/mL TUNI, the growth inhibition rate of the wild-type strain was significantly lower than that of Δ*Mosec61β*. Similarly, under 5.0 mM DTT treatment, the growth inhibition rate showed the same differences between the wild-type and Δ*Mosec61β* strains (Figure 8(b)). The growth of the Δ*Mosec61β* strain was inhibited by ER stress factors.

In response to ER stress, ER-mediated autophagy (ER-phagy) occurs to maintain the normal function of the ER. Therefore, we hypothesized that ER-phagy of Δ*Mosec61β* is impaired under ER stress. MoSec63-GFP is an integral ER membrane protein. It has been reported that 5 μM DTT can induce MoSec63-GFP cleavage, consistent with the activation of selective autophagy by ER stress. We constructed the MoSec63-GFP fluorescent vector and transformed it into the wild-type Guy11 strain and the Δ*Mosec61β* mutant strain. Under the induction of DTT, the expression of GFP differed significantly between Guy11 and Δ*Mosec61β* (Figure 8(c)). At 0 h, there was little free GFP in Guy11 and more free GFP in Δ*Mosec61β*. At 4 h, the amount of GFP-containing fragments was substantially enhanced in Δ*Mosec61β* compared with Guy11 (Figure 8(c)). These results indicate that the absence of MoSec61β stimulates ER-mediated autophagy.

## Discussion

In eukaryotic cells, protein translocation can occur co- or post-translationally, depending on the hydrophobicity of the precursor protein. These mechanisms require the heterotrimeric Sec61 complex for transmembrane transport [28]. In this study, we reported the biofunctions of Sec61β in *M. oryzae*. By homology alignment, we found only one gene encoding the Sec61β protein in rice blast fungus, MGG_03644, and we named that gene *MoSEC61β*. Loss of *MoSEC61β* had no apparent influence on conidial germination or appressorium formation but showed significant pathogenesis defects. During infection, Δ*Mosec61β* showed restricted IH and caused a host ROS burst that induced rice innate immunity because of impaired effector secretion.

Although Sec61β has not been studied in other plant pathogenic fungi, the maintenance function of ER under stress has been widely researched. As an essential organelle in cells, the ER keeps continuous renovation to maintain its function and integrity when faced with changes in the external and internal environment [29]. The ER performs quality control through the unfolded protein response (UPR), which leads to the upregulation of chaperone, decrease of protein synthesis and reverse transport of misfolded protein to the cytosol for degradation [30]. In *M. oryzae*, a double knockout strain for ERAD (ER-associated degradation, ERAD) genes, Δ*Mohrd*Δ*Moder1*, lost its pathogenicity. Compared with the wild-type strain, Δ*Mohrd*Δ*Moder1* shows an unfolded protein response under normal conditions, and the secretion of pathogenic effector proteins is affected [31]. Another study found that ERQC (ER glycoprotein folding quality control) components, such as Sgls1, Gls2, and Gtb1, were N-glycosylated and were involved in the process of mycelial growth, conidiation, and invasive hyphal growth in host cells [32]. Similarly, the ER chaperone protein Lhs1 is also involved in rice infection in the blast fungus. The UPR target genes, including *SIL1, KAR2, PDI1*, and *SCJ1*, are upregulated, and the function and secretion of AVR-Pita in Δ*lhs1* are impaired [33]. All these results indicate the importance of ER homeostasis for protein secretion and pathogenicity of rice blast.

During plant-pathogen interactions, pathogens produce effectors to resist and escape the immune response of plants. Our results found that loss of *MoSEC61β* impacts the localization of the apoplastic effectors Bas4 and Slp1 and thus faces a strong plant immune response, including defects in ROS scavenging and callose degradation, and high expression of *PR1a*. Accordingly, in heat-killed plant cells, the growth of the invasive hypha of Δ*Mosec61β* was not affected. This result is in keeping with the fact that the secretion of apoplastic effectors is transported via the ER-to-Golgi pathway. We thus conclude that unlike in yeast, where Sec61β is not necessary, in *M. oryzae*, the β subunit is necessary for the transposon function of the Sec61 complex. A previous study also suggested other roles played by Sec61β in addition to its function as a component of the translocon. In yeast, Sec61β was found to interact with Sec4p and the exocyst complex component, including Sec8p and Sec15p [34]. It is well-known that the transport of cytoplasmic effectors is mediated by the exocyst complex. Loss of the exocyst components Exo70 and Sec5 causes defects in proper secretion of the cytoplasmic effector Pwl2 [10]. We therefore investigated whether the loss of MoSec61β in *M. oryzae* would also influence the ability of exocysts and ultimately affect the function of cytoplasmic effectors. We observed the localization of Pwl2 during Δ*Mosec61β* invasion and found similar localization of Pwl2 between the Δ*Mosec61β* and wild-type strains. These results showed that the β subunit is necessary for the transposon function of the Sec61 complex and that the absence of Sec61β did not affect the normal function of exocysts in the rice blast fungus.

In addition to the defects observed during the infection process, we also found that the mutant exhibited deficiencies in its ability to infect the host. Although Δ*Mosec61β* forms appressoria with normal morphology, the turgor pressure of the appressoria is generally low and cannot successfully penetrate the host surface. The maturation of the appressorium requires the mediation of core autophagy. Autophagy helps the appressorium to produce glycerol and turgor pressure by transporting and degrading inclusions inside the conidia (mainly glycogen and lipid droplets) [35]. In our experiments, we also found that the normal degradation of glycogen and lipid droplets in the mutant was inhibited, suggesting dysregulated autophagy. However, both the nuclear degradation and GFP-MoAtg8 degradation tests indicated that autophagy can occur normally in Δ*Mosec61β*.

As the channel connecting the ER to the cytoplasm, Sec61 also plays a role in ERAD, mediating the secretion of misfolding proteins in the ER back into the cytoplasm [36]. It is possible that the dysfunction of the Sec61 complex inhibits the ERAD process, thereby causing the accumulation of misfolding proteins. In fact, except for ERAD, which degrades proteins through the ubiquitin-proteasome system, ER also employs the specified autophagy ER-phagy to maintain its inner balance [37]. Therefore, we utilized the ER membrane protein Sec63-GFP to monitor the ER-phagy level inside Δ*Mosec61β*. Our results showed that under normal conditions, Sec63 is expressed at a higher level in Δ*Mosec61β*. After 4 h of treatment with DTT to induce ER stress, the degradation of Sec63-GFP was also more significant, indicating a higher level of ER-phagy in Δ*Mosec61β* than in the wild-type Guy11 strain. We hypothesized that Δ*Mosec61β* requires a stronger level of ER-mediated autophagy to compensate for the restriction of ERAD.

In summary, our results reveal that MoSec61β is necessary for the vegetative growth, asexual development, appressorium penetration, plant immunity evasion and pathogenesis of rice blast fungus. MoSec61β plays pivotal roles in turgor pressure by influencing the mobilization and degradation of glycogen and lipids. Deletion of MoSec61β and *M. oryzae* is deficient in overcoming plant defense responses. MoSec61β controls pathogenicity via the secretion of the apoplastic effector Bas4 and Slp1. In addition, MoSec61β is involved in ER-phagy in response to ER stress.

## Materials and methods

### Strains and culture conditions

The *Magnaporthe oryzae* strain Guy11 and derivative transformants were cultured on complete medium (CM) in a growth chamber at 25 or 28°C with a 16 h light and 8 h dark cycle [38]. Target gene replacement method was used to generate null mutants (Fig. S8, Table S2). For the oxidative stress test, the strains were cultured on CM media with 5.0 mM H_2_O_2_ and cultured in dark at 28°C. For cell wall integrity tests, the strains were cultured on minimal medium (MM) medium supplemented with 0.0025% SDS, 100 μg/mL CFW and 50 μg/mL CR in dark at 28°C. For ER stress tests, the strains were cultured on MM with 0.5 μg/ml tunicamycin (TUNI) and 5.0 mM dithiothreitol (DTT) and cultured under a 16 h light and 8 h dark cycle at 25°C.

### Phenotypic characterization

For fungal growth and conidiation assays, a 6 × 6 mm mycelium plug of *M. oryzae* strains was inoculated on complete medium (CM) under the conditions of 25°C, 16 h light/8 h dark for 8 days. The diameter was measured after 8 days. Conidiation was detected by the strains grown for 8 days. The spores were washed with 3 mL water, filtered and diluted to 2 mL. The number of conidia was determined by a counter. To measure conidial germination and appressorium formation, 20 μL of spore suspension (5 × 10^4^ conidia/mL) was dropped onto plastic coverslips and incubated at 22°C. Conidial germination and appressorium formation were observed for 4–24 h hpi. To observe conidiophore development of *M. oryzae* strains, vegetative hyphae inside the medium were sliced into thin pieces, and the pieces were cultured under 16 h light/8 h dark for 24 h at 25°C [39].

### Pathogenicity and plant infection assays

Two-week-old seedlings of rice (*Oryza sativa* cv CO-39) were used in plant infection assays. Conidia harvested from 8-day-old growth on CM plates were resuspended in 0.2% (w/v) gelatin solution for a concentration of 1 × 10^5^ conidia/mL. The suspension was sprayed evenly onto rice leaves using an artist’s airbrush (Badger Co., Franklin Park, Illinois). The inoculated plants were placed in a dew chamber at 25°C for 48 h in the dark and then transferred to a growth chamber with a cycle of 16 h of light and 8 h of dark using fluorescent lights. The plants were examined for lesions 7 days after inoculation. Disease severity was rated with the scale developed.

Infection assays were carried out three times for host penetration assays, and leaf segments were excised from 8-day-old seedlings of barley (*Hordeum vulgare* cv ZJ-8). Drops (20 μL) of conidial suspension (5 × 10^4^ conidia/mL) were deposited onto the upper surface of the excised leaves in a dew chamber at 25°C. The leaves were examined for disease lesions at 24, 48, 72, and 96 h after inoculation after decoloration by methanol and preservation in lactophenol, as described previously [40].

### Polarity growth and CFW staining

Sterilized coverslips were inserted obliquely on the CM solid medium, a suitable amount of mycelium was picked with a toothpick and inoculated onto the CM plates with coverslips, and strains were cultured in a constant temperature incubator at 25°C under 16 h light and 8 h dark. When the aerial hyphae of the strain grew to the middle of the cover glass, the cover glass was gently extracted from the CM plate and placed under a microscope to observe the polarity growth of the strain. CFW solution was added dropwise to the coverslip with aerial hyphae, and after staining for 5 min, it was placed under a fluorescence microscope for observation and photograph recording. The experiment was repeated three times.

### Incipient cytorrhysis assays

Incipient cytorrhysis assays were used to determine the appressorium turgor [41]. In a humid environment, drops (20 μL) of conidial suspension (1 × 10^5^ conidia/mL) were incubated on plastic coverslips for 24 h. After that step, the water was carefully removed and replaced with an equal volume of a glycerol solution with a concentration ranging from 0.5 M to 1.5 M. After 1 min of incubation in glycerol solution, the number of appressoria that had collapsed was recorded. The experiments were repeated three times, and more than 200 appressoria were observed each time.

### Rice leaf sheath assays

The fresh spore suspension (1 × 10^5^ conidia/mL) was injected into the leaf sheath by a disposable syringe (without bubbles). The leaf-sheath tube filled with spore suspension was cultured in an incubator at 25°C with a light cycle of 16 h and darkness of 8 h. Then, the transparent thin layer of the leaf sheath was carefully cut off with a blade, and the surface of the leaf sheath in contact with the spores was turned upward to make a slide of the leaf sheath for fluorescence microscope observation. The experiment was repeated three times.

### Lipid droplets and glycogen staining

Tricyclazole (1 μL of 10 μg/mL to inhibit the formation of melanin of the attached cells and reduce the influence on the experimental observation) was added to 1 mL (1 × 10^5^ conidia/mL) spore suspension. Drops (20 μL) of the spore suspension were inoculated on a hydrophobic membrane at 25°C in the dark and moisturized for 0 h, 8 h, 16 h, and 24 h, respectively. The water was carefully removed and replaced with an equal volume of a Nile red staining solution. After 30 min of incubation in Nile red staining solution, the transport and degradation of red lipid droplets were observed under a fluorescence microscope. Similarly, glycogen was stained in I_2_/KI solution for 1 min. The experiment was repeated 3 times, and 200 conidia were counted for each treatment.

### Callose deposition staining

For callose deposition staining, leaves of barley (at 32hpi) were fixated and destained in 1:3 acetic acid/ethanol, the saturated destaining solution was replaced until the material was transparent (usually overnight). Fixated and destained leaves or seedlings were washed in 150 mM K_2_HPO_4_ for 30 min. Leaves of barley were incubated for at least 2 h in 150 mM K_2_HPO_4_ and 0.01% aniline blue (staining solution) in a 2 mL tube wrapped in aluminum foil for light protection.

### RNA preparation and RT-PCR analysis

Fresh plant leaves (0.4 g) which inoculated with spore suspension were ground to powder with liquid nitrogen, and RNA was extracted using RNAiso Plus (TaKaRa, Japan) according to manufacturer instructions. Reverse transcription of total RNA was carried out using PrimeScrip^TM^ RT regent Kit with gDNA Eraser (Takara, Japan). The qRT-PCR was then performed using TB Green® Premix EX Taq^TM^ (Tli RNaseH Plus) (TaKaRa, Japan) to analyze the expression level of plant defense-related genes (*PR1a, PBZ1*) and callose synthase-encoding genes (*OsGSL1, OsGSL3, OsGSL5*).

### Western blot analysis

For Sec63-GFP and cleaved GFP assays, the Sec63-GFP vector was transformed into the Guy11 and mutant strains using ATMT. The Sec63-GFP-expressing strains were grown in CM medium for 7 days and then shifted to nitrogen starvation (SD-N) medium with 5 μM DDT for 4 h to induce ER-phagy. Total proteins were separated on SDS-PAGE gels and transferred to polyvinylidene diﬂuoride membranes for Western blot analysis. Sec63-GFP was detected with a primary anti-GFP antibody (GFP-Tag Rabbit mAb, Huabio, Hangzhou, China) and a secondary antibody (goat anti-rabbit IgG HRP, Beyotime, Shanghai, China). GAPDH antibodies (Huabio, Hangzhou, China) were used to conﬁrm equal protein loading of each strain.

## Supplementary Material

Supplemental Material

## References

[1] Couch BC, Kohn LM. A multilocus gene genealogy concordant with host preference indicates segregation of a new species, Magnaporthe oryzae, from M. grisea. Mycologia. 2002;94:683–693.PMID:21156541. 21156541 10.1080/15572536.2003.11833196

[2] Dean RA. Signal pathways and appressorium morphogenesis. Annu Rev Phytopathol. 1997;35:211–234.PMID:15012522. 15012522 10.1146/annurev.phyto.35.1.211

[3] Thines E, Weber RW, Talbot NJ. MAP kinase and protein kinase A-dependent mobilization of triacylglycerol and glycogen during appressorium turgor generation by Magnaporthe grisea. Plant Cell. 2000;12:1703–1718.PMID:11006342. 11006342 10.1105/tpc.12.9.1703 PMC149080

[4] Peng Y, van Wersch R, Zhang Y. Convergent and divergent signaling in PAMP-triggered immunity and effector-triggered immunity. Mol Plant Microbe Interact. 2018;31:403–409.PMID:29135338. 29135338 10.1094/MPMI-06-17-0145-CR

[5] Medzhitov R, Janeway CJ. Innate immunity: the virtues of a nonclonal system of recognition. CELL. 1997;91:295–298.PMID:9363937. 9363937 10.1016/s0092-8674(00)80412-2

[6] Zhou Z, Pang Z, Li G, et al. Endoplasmic reticulum membrane-bound MoSec62 is involved in the suppression of rice immunity and is essential for the pathogenicity of Magnaporthe oryzae. Mol Plant Pathol. 2016;17:1211–1222.PMID:26679839. 26679839 10.1111/mpp.12357 PMC6638330

[7] Wang C, Liu Y, Liu L, et al. The biotrophy-associated secreted protein 4 (BAS4) participates in the transition of Magnaporthe oryzae from the biotrophic to the necrotrophic phase. Saudi J Biol Sci. 2019;26:795–807.PMID:31049006. 31049006 10.1016/j.sjbs.2019.01.003 PMC6486625

[8] Kankanala P, Czymmek K, Valent B. Roles for rice membrane dynamics and plasmodesmata during biotrophic invasion by the blast fungus. Plant Cell. 2007;19:706–724.PMID:17322409. 17322409 10.1105/tpc.106.046300 PMC1867340

[9] Khang CH, Berruyer R, Giraldo MC, et al. Translocation of Magnaporthe oryzae effectors into rice cells and their subsequent cell-to-cell movement. Plant Cell. 2010;22:1388–1403.PMID:20435900. 20435900 10.1105/tpc.109.069666 PMC2879738

[10] Giraldo MC, Dagdas YF, Gupta YK, et al. Two distinct secretion systems facilitate tissue invasion by the rice blast fungus Magnaporthe oryzae. Nat Commun. 2013;4:1996.PMID:23774898. 23774898 10.1038/ncomms2996 PMC3709508

[11] Wang Y, Wang Y. Trick or treat: microbial pathogens evolved apoplastic effectors modulating plant susceptibility to infection. Mol Plant Microbe Interact. 2018;31:6–12.PMID:29090656. 29090656 10.1094/MPMI-07-17-0177-FI

[12] Mentlak TA, Kombrink A, Shinya T, et al. Effector-mediated suppression of chitin-triggered immunity by magnaporthe oryzae is necessary for rice blast disease. Plant Cell. 2012;24:322–335.PMID:22267486. 22267486 10.1105/tpc.111.092957 PMC3289562

[13] Viotti C. ER to golgi-dependent protein secretion: the conventional pathway. Methods Mol Biol. 2016;1459:3–29.PMID:27665548. 27665548 10.1007/978-1-4939-3804-9_1

[14] Nie H, Zhang L, Zhuang H, et al. Secreted protein MoHrip2 is required for full virulence of Magnaporthe oryzae and modulation of rice immunity. Appl Microbiol Biotechnol. 2019;103:6153–6167.PMID:31154490. 31154490 10.1007/s00253-019-09937-2

[15] Zhang S, Xu JR. Effectors and effector delivery in Magnaporthe oryzae. PLOS Pathog. 2014;10:e1003826.PMID:24391496. 24391496 10.1371/journal.ppat.1003826 PMC3879361

[16] Polgar N, Lee AJ, Lui VH, et al. The exocyst gene Sec10 regulates renal epithelial monolayer homeostasis and apoptotic sensitivity. Am J Physiol Cell Physiol. 2015;309:C190–201.PMID:26040895. 26040895 10.1152/ajpcell.00011.2015 PMC4525081

[17] Bebok Z, Mazzochi C, King SA, et al. The mechanism underlying cystic fibrosis transmembrane conductance regulator transport from the endoplasmic reticulum to the proteasome includes Sec61beta and a cytosolic, deglycosylated intermediary. J Biol Chem. 1998;273:29873–29878.PMID:9792704. 9792704 10.1074/jbc.273.45.29873

[18] Mothes W, Prehn S, Rapoport TA. Systematic probing of the environment of a translocating secretory protein during translocation through the ER membrane. Embo J. 1994;13:3973–3982. PMID:8076593. 8076593 10.1002/j.1460-2075.1994.tb06713.x PMC395317

[19] Esnault Y, Blondel MO, Deshaies RJ, et al. The yeast SSS1 gene is essential for secretory protein translocation and encodes a conserved protein of the endoplasmic reticulum. Embo J. 1993;12:4083–4093. PMID:8223425. 8223425 10.1002/j.1460-2075.1993.tb06092.x PMC413701

[20] Feng D, Zhao X, Soromani C, et al. The transmembrane domain is sufficient for Sbh1p function, its association with the Sec61 complex, and interaction with Rtn1p. Journal of Biological Chemistry. 2007;282(42):30618–30628.PMID:17699516. 10.1074/jbc.M701840200 PMC2361393 17699516

[21] Kelkar A, Dobberstein B. Sec61beta, a subunit of the Sec61 protein translocation channel at the endoplasmic reticulum, is involved in the transport of Gurken to the plasma membrane. Bmc Cell Biol. 2009;10:11.PMID:19226464. 19226464 10.1186/1471-2121-10-11 PMC2653466

[22] Zhang WJ, Hanisch S, Kwaaitaal M, et al. A component of the Sec61 ER protein transporting pore is required for plant susceptibility to powdery mildew. Front Plant Sci. 2013;4:127.PMID:23720664. 23720664 10.3389/fpls.2013.00127 PMC3655284

[23] Wang ZY, Soanes DM, Kershaw MJ, et al. Functional analysis of lipid metabolism in Magnaporthe grisea reveals a requirement for peroxisomal fatty acid beta-oxidation during appressorium-mediated plant infection. Mol Plant Microbe Interact. 2007;20:475–491.PMID:17506326. 17506326 10.1094/MPMI-20-5-0475

[24] Li R, Jia Z, Trush MA. Defining ROS in biology and medicine. React Oxyg Species (Apex). 2016;1:9–21. PMID:29707643. 29707643 10.20455/ros.2016.803 PMC5921829

[25] Nakashita H, Yoshioka K, Takayama M, et al. Characterization of PBZ1, a probenazole-inducible gene, in suspension-cultured rice cells. Biosci Biotechnol Biochem. 2001;65:205–208.PMID:11272832. 11272832 10.1271/bbb.65.205

[26] Wang C, Li C, Duan G, et al. Overexpression of Magnaporthe oryzae systemic defense trigger 1 (MoSDT1) confers improved rice blast resistance in rice. Int J Mol Sci. 2019;20. PMID:31557947. DOI:10.3390/ijms20194762. PMC6802482 31557947

[27] Luna E, Pastor V, Robert J, et al. Callose deposition: a multifaceted plant defense response. Mol Plant Microbe Interact. 2011;24:183–193.PMID:20955078. 20955078 10.1094/MPMI-07-10-0149

[28] Lang S, Pfeffer S, Lee PH, et al. An update on Sec61 channel functions, mechanisms, and related diseases. Front Physiol. 2017;8:887.PMID:29163222. 29163222 10.3389/fphys.2017.00887 PMC5672155

[29] Chino H, Mizushima N. ER-Phagy: quality control and turnover of endoplasmic reticulum. Trends Cell Biol. 2020;30:384–398.PMID:32302550. 32302550 10.1016/j.tcb.2020.02.001

[30] Hwang J, Qi L. Quality control in the endoplasmic reticulum: crosstalk between ERAD and UPR pathways. Trends Biochem Sci. 2018;43:593–605.PMID:30056836. 30056836 10.1016/j.tibs.2018.06.005 PMC6327314

[31] Tang W, Jiang H, Aron O, et al. Endoplasmic reticulum-associated degradation mediated by MoHrd1 and MoDer1 is pivotal for appressorium development and pathogenicity of Magnaporthe oryzae. Environ Microbiol. 2020. PMID:32410295. DOI:10.1111/1462-2920.15069. 32410295

[32] Chen XL, Liu C, Tang B, et al. Quantitative proteomics analysis reveals important roles of N-glycosylation on ER quality control system for development and pathogenesis in Magnaporthe oryzae. Plos Pathog. 2020;16:e1008355.PMID:32092131. 32092131 10.1371/journal.ppat.1008355 PMC7058352

[33] Yi M, Chi MH, Khang CH, et al. The ER chaperone LHS1 is involved in asexual development and rice infection by the blast fungus Magnaporthe oryzae. Plant Cell. 2009;21:681–695.PMID:19252083. 19252083 10.1105/tpc.107.055988 PMC2660637

[34] Toikkanen JH, Miller KJ, Soderlund H, et al. The beta subunit of the Sec61p endoplasmic reticulum translocon interacts with the exocyst complex in Saccharomyces cerevisiae. J Biol Chem. 2003;278:20946–20953.PMID:12665530. 12665530 10.1074/jbc.M213111200

[35] Liu XH, Lu JP, Zhang L, et al. Involvement of a Magnaporthe grisea serine/threonine kinase gene, MgATG1, in appressorium turgor and pathogenesis. Eukaryot Cell. 2007;6:997–1005.PMID:17416896. 17416896 10.1128/EC.00011-07 PMC1951528

[36] Romisch K. Endoplasmic reticulum-associated degradation. Annu Rev Cell Dev Biol. 2005;21:435–456.PMID:16212502. 16212502 10.1146/annurev.cellbio.21.012704.133250

[37] Bernales S, Schuck S, Walter P. ER-phagy: selective autophagy of the endoplasmic reticulum. Autophagy. 2007;3:285–287.PMID:17351330. 17351330 10.4161/auto.3930

[38] Talbot NJ, Ebbole DJ, Hamer JE. Identification and characterization of MPG1, a gene involved in pathogenicity from the rice blast fungus Magnaporthe grisea. Plant Cell. 1993;5:1575–1590.PMID:8312740. 8312740 10.1105/tpc.5.11.1575 PMC160387

[39] Cao H, Huang P, Zhang L, et al. Characterization of 47 Cys2 -His2 zinc finger proteins required for the development and pathogenicity of the rice blast fungus Magnaporthe oryzae. New Phytol. 2016;211:1035–1051.PMID:27041000. 27041000 10.1111/nph.13948

[40] Kim S, Ahn IP, Rho HS, et al. MHP1, a Magnaporthe grisea hydrophobin gene, is required for fungal development and plant colonization. Mol Microbiol. 2005;57:1224–1237.PMID:16101997. 16101997 10.1111/j.1365-2958.2005.04750.x

[41] Howard RJ, Ferrari MA, Roach DH, et al. Penetration of hard substrates by a fungus employing enormous turgor pressures. Proc Natl Acad Sci USA. 1991;88:11281–11284.PMID:1837147. 1837147 10.1073/pnas.88.24.11281 PMC53118

