# Supplementary material for: MoSec61β, the beta subunit of Sec61, is involved in fungal development and pathogenicity, plant immunity, and ER-phagy in Magnaporthe oryzae
Source: Virulence. 2020 Nov 29;11(1):1685–700. doi: 10.1080/21505594.2020.1848983 (PMC7714445; doi:10.1080/21505594.2020.1848983)
Supplement: Supplemental Material [file KVIR_A_1848983_SM5734.zip › captions.docx]

**Fig. S1** Multiple sequence alignment of MoSec61β homolog proteins using CLC Main Workbench.

**Fig. S2** The growth inhibition rate of strains to 0.0025% SDS, 100 μg/mL CFW, and 50 μg/mL Congo red (CR). Error bars represent the standard deviation. Significant differences compared with the wild-type strain were estimated by Duncan’s test: **P < 0.01, *P < 0.05.

**Fig. S3** Mycelium disks of Guy11, Δ*Mosec61β*, and *Mosec61β*c were inoculated on barley leaves for 4 days.

**Fig. S4** Morphology of conidia and appressoria in Guy11 and Δ*Mosec61β*. Bar = 20 μm.

**Fig. S5** The transcriptional expression of *PR1a* and *PBZ1* in the infected rice was analyzed at different time. Error bars represent the standard deviation.

**Fig. S6** Nuclear degradation in Guy11 and Δ*Mosec61*β. Error bars represent the standard deviation.

**Fig. S7** GFP-MoAtg8 proteolysis assay. Total proteins were extracted from GFP-MoAtg8-expressing transformants exposed to nitrogen starvation conditions for 0 and 4 h. Full-length GFP-MoAtg8 and free GFP were detected using GFP antibodies as described in the Materials and Methods. The extent of autophagy was estimated by calculating the amount of free GFP compared with the total amount of intact GFP-MoAtg8 and free GFP. Quantitative analysis of the individual bands was performed using ImageJ software.

**Fig. S8** Target gene replacement of the indicated genes. PCR using a unique recombinational DNA fragment was used to verify the mutant knockout event. An ~1.0-2.0 kb band was detected in the null mutants on an electrophoretic gel. In contrast, the wild-type strain was not detected (Upper). The mutants were verified by double PCR for the targeted gene using the β-tubulin gene as a positive control (~0.25 kb band). The wild-type strain Guy11 produced a characteristic band, indicating the targeted gene (~0.3 kb band), while the null mutants did not (Lower).

**Table S1.** Characteristics of the wild-type Guy11, the Δ*Mosec61* mutant, and complementation strain *Mosec61βc.*

**Table S2.** Primers used in this study.
