# Supplementary material for: MoSec61β, the beta subunit of Sec61, is involved in fungal development and pathogenicity, plant immunity, and ER-phagy in Magnaporthe oryzae
Source: Virulence. 2020 Nov 29;11(1):1685–700. doi: 10.1080/21505594.2020.1848983 (PMC7714445; doi:10.1080/21505594.2020.1848983)
Supplement: Supplemental Material [file KVIR_A_1848983_SM5734.zip › Table S2.docx]

**Table S2 Primers used in this study.**

| **Primer name** | **Primer sequence (5’-3’)** |
| --- | --- |
| MoSEC61β-UP-F  MoSEC61β-UP-R  MoSEC61β-Down-F  MoSEC61β-Down-R  HPH-F  HPH-R  MoSEC61β-L-F  MoSEC61β-S-F  MoSEC61β-S-R  TUBLIN-F  TUBLIN-R  MoSEC61β-C-F  MoSEC61β-C-R  ACTIN-RT-F | GGTACCCGGGGATCCTCTAGATCTCTTCGTGGCATTCCT  TTCATTGTTGACCTCCACTATGTCTCTTATTCGCAACTGA  GCAAAGGAATAGAGTAGATGAATGATTGTCAGGTCAGGTT  ACGACGGCCAGTGCCAAGCTTGCCGTCTCCAGTCTCTAT  TAGTGGAGGTCAACAATGAATG  CATCTACTCTATTCCTTTGCCC  CAATAGATTGCGAGCAGC  GACGACACTCTCCTAACG  CAATAACTGGCGATGATGG  TTCCGCGCTGTCACCGTTCC  GGGCCTCCTCCTCGTACTCCTCTT  CAATCACAATGGCCGGATCC ATGGTTAGTCAAGCTTACTC  CCCTTGCTCACCATCCCGGGGCCGCCGCCGCCGCCGGGGCCACAGCGGTTCTCCAACACAATGGTTCGGGTATGTGC |
| ACTIN-RT-R | CGACAATGGACGGGAAGZC |
| HPH-RT-F | ATGTCCTGCGGGTAAATAGC |
| HPH-RT-R | GATGCAATAGGTCAGGCTCTC |
| TUBLIN-RT-F | ACAACTTCGTCTTCGGTCAG |
| TUBLIN-RT-R  PR1A-RT-F  PR1A-RT-R  PBZ1-RT-F  PBZ1-RT-R  OsGSL1-RT-F  OsGSL1-RT-R  OsGSL3-RT-F  OsGSL3-RT-R  OsGSL5-RT-F  OsGSL5-RT-R  Actin-RT-F  Actin-RT-R | GTGATCTGGAAACCCTGGAG  GGTGTCGGAGAAGCAGTGGTA  GCGAGTAGTTGCAGGTGATGAAG  GGCTTGGTCGACGACATTG  CAGGGTTAAGCTTCATGGTGTAGA  TGAGGACCTGCCACGATT  CACGCTGATTGCGAACAT  TGGCAAGCGACCACATAG  AGACCTTAGCACGGACTG  GTGGTGTCCCTGCTATGA  GTTGTTTGCTATTCTCCC  GAGCTACGAGCTTCCTGATGGA  CCTCAGGGCAGCGGAAA |
