# Supplementary material for: MoSec61β, the beta subunit of Sec61, is involved in fungal development and pathogenicity, plant immunity, and ER-phagy in Magnaporthe oryzae
Source: Virulence. 2020 Nov 29;11(1):1685–700. doi: 10.1080/21505594.2020.1848983 (PMC7714445; doi:10.1080/21505594.2020.1848983)
Supplement: Supplemental Material [file KVIR_A_1848983_SM5734.zip › Table S1 .docx]

**Table S1 Characteristics of wild-type Guy11, Δ*Mosec61* and complementation strain *Mosec61βc***

| strain | Conidium and appressorium morphology and function |
| --- | --- |
|  | Conidial germination at 4 h (%) |
| Guy11 | 99.71±0.10a |
| Δ*Mosec61β* | 97.98±1.31a |
| *Mosec61βc* | 99.11±1.45a |
|  | Appressorium formation at 24 h (%) |
| Guy11 | 95.83±1.74a |
| Δ*Mosec61β* | 95.33±1.85a |
| *Mosec61βc* | 94.81±2.08a |
|  | The diameter of appressorium (μm) |
| Guy11 | 9.91±1.26 a |
| Δ*Mosec61β* | 9.96±0.68 a |
| *Mosec61βc* | 9.85±1.12 a |

Average values with the same lowercase letters in each column are not significantly different, as estimated by the Duncan’s test (SSR).
