# Supplementary figures and images for: MoSec61β, the beta subunit of Sec61, is involved in fungal development and pathogenicity, plant immunity, and ER-phagy in Magnaporthe oryzae
Source: Virulence. 2020 Nov 29;11(1):1685–700. doi: 10.1080/21505594.2020.1848983 (PMC7714445; doi:10.1080/21505594.2020.1848983)

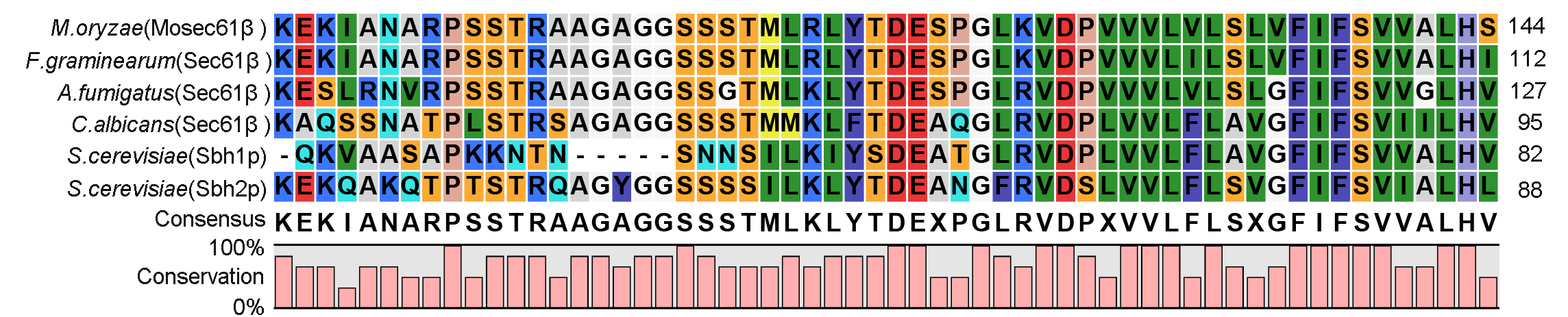

Supplement: Supplemental Material [file KVIR_A_1848983_SM5734.zip › Figure S1.jpg]

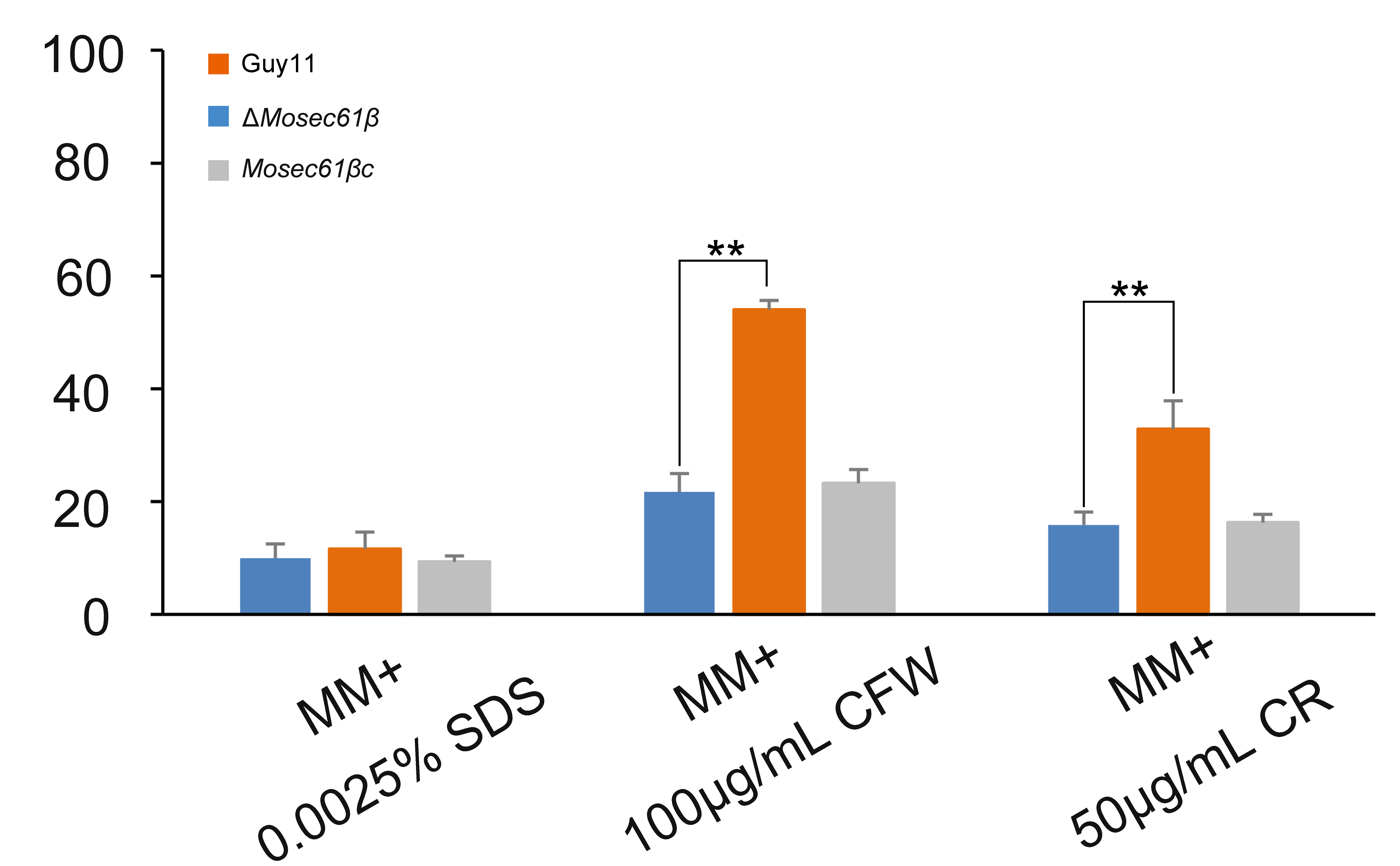

Supplement: Supplemental Material [file KVIR_A_1848983_SM5734.zip › Figure S2.jpg]

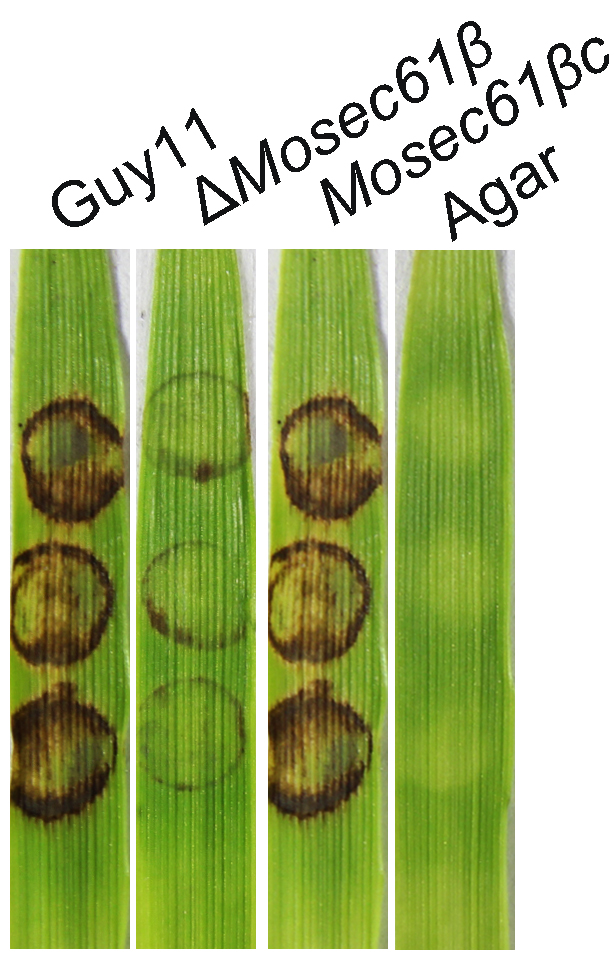

Supplement: Supplemental Material [file KVIR_A_1848983_SM5734.zip › Figure S3.jpg]

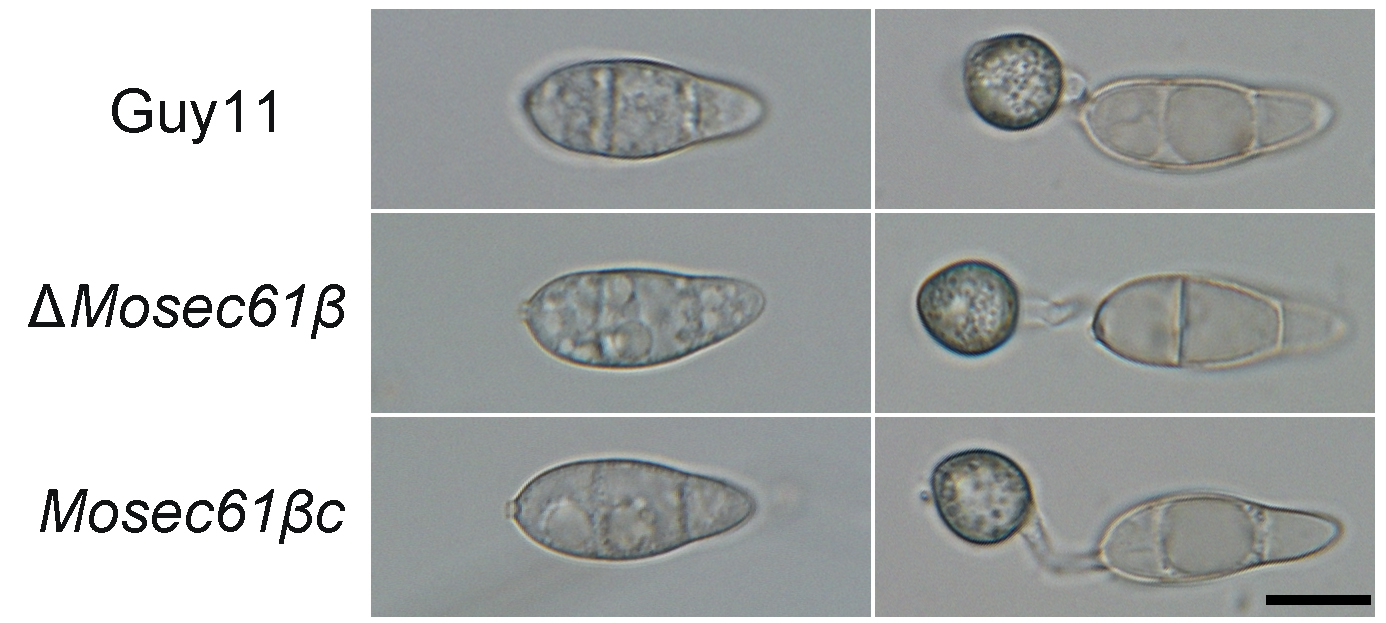

Supplement: Supplemental Material [file KVIR_A_1848983_SM5734.zip › Figure S4.jpg]

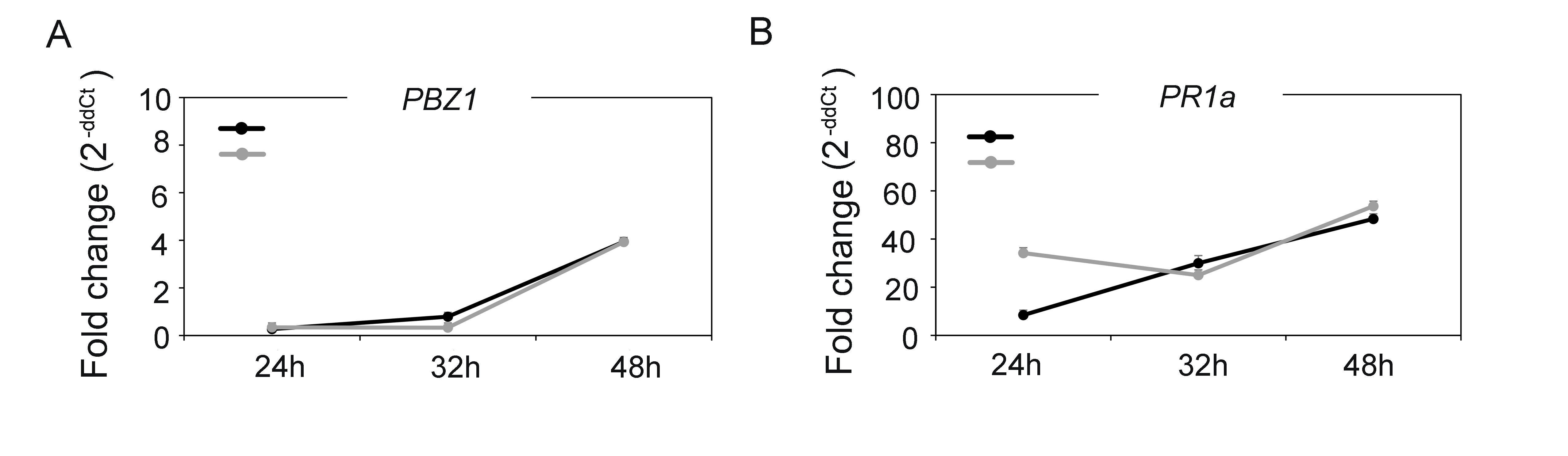

Supplement: Supplemental Material [file KVIR_A_1848983_SM5734.zip › Figure S5.jpg]

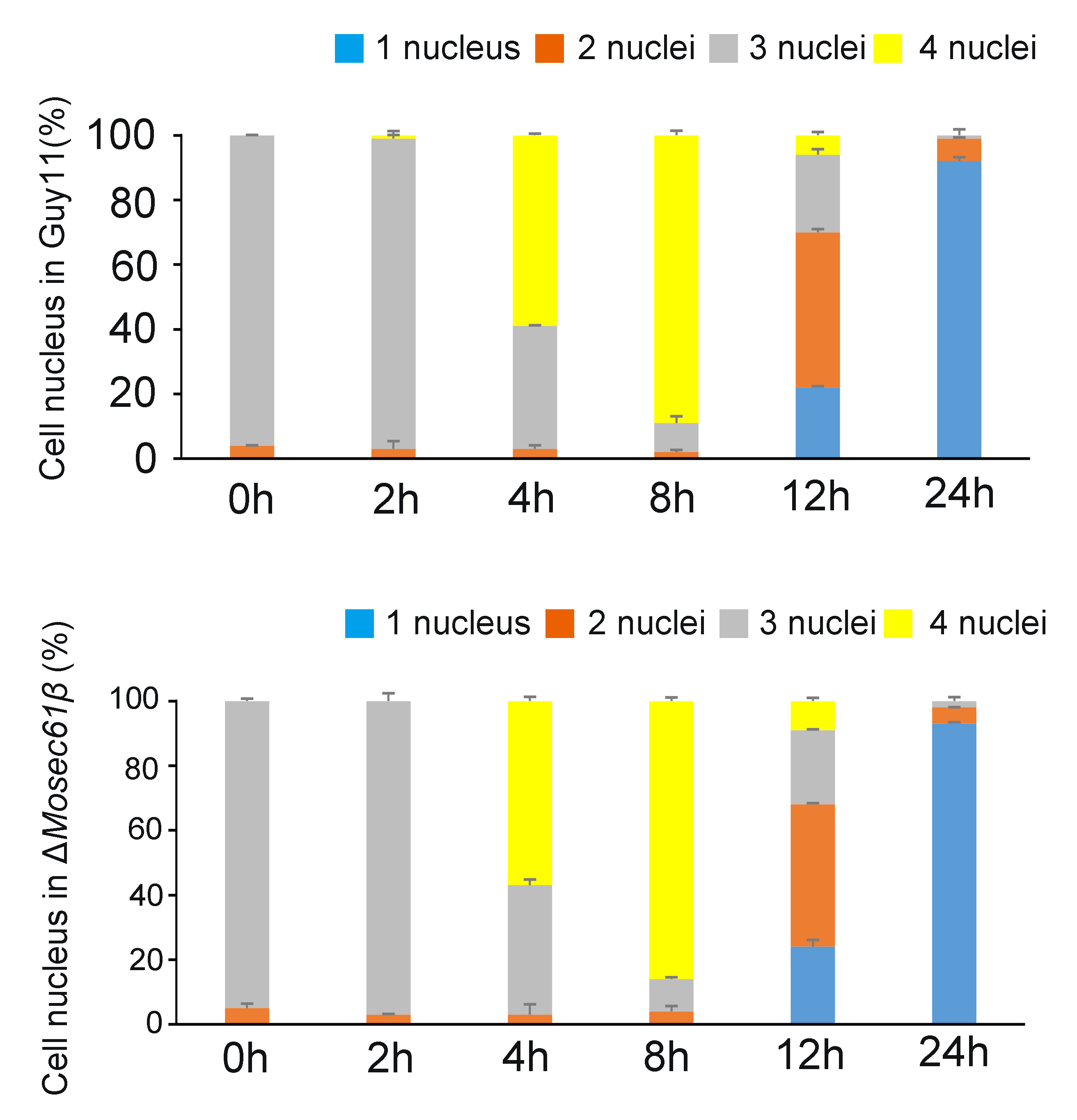

Supplement: Supplemental Material [file KVIR_A_1848983_SM5734.zip › Figure S6.jpg]

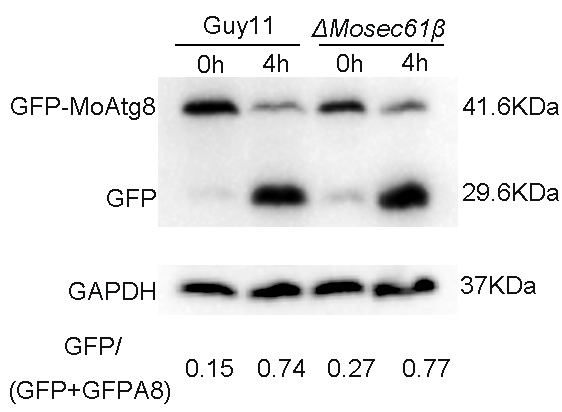

Supplement: Supplemental Material [file KVIR_A_1848983_SM5734.zip › Figure S7.jpg]

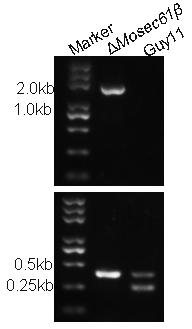

Supplement: Supplemental Material [file KVIR_A_1848983_SM5734.zip › Figure S8.jpg]
